# Genetic homogeneity of *Anopheles maculatus* in Indonesia and origin of a novel species present in Central Java

**DOI:** 10.1186/s13071-019-3598-1

**Published:** 2019-07-15

**Authors:** Triwibowo Ambar Garjito, Umi Widiastuti, Mujiyono Mujiyono, Mega Tyas Prihatin, Widiarti Widiarti, Riyani Setyaningsih, Siti Alfiah, Barandi Sapta Widartono, Din Syafruddin, Tri Baskoro Tunggul Satoto, Laurent Gavotte, Michael J. Bangs, Sylvie Manguin, Roger Frutos

**Affiliations:** 1 Institute for Vector and Reservoir Control Research and Development, National Institute of Health Research and Development, The Ministry of Health of Indonesia, Salatiga, Central Java Indonesia; 2 University of Montpellier, Montpellier, France; 3 HydroSciences Montpellier (UMR-HSM), Institut de Recherche pour le Développement (IRD France), CNRS, Montpellier, France; 4 Department of Geographical Information System, Faculty of Geography, Gadjah Mada University, Yogyakarta, Indonesia; 5 Eijkman Institute for Molecular Biology, Jakarta, Indonesia; 6 Department of Parasitology, Faculty of Medicine, Public Health and Nursing, Gadjah Mada University, Yogyakarta, Indonesia; 7 ISEM, University of Montpellier, Montpellier, France; 8 Public Health & Malaria Control, International SOS/PT. Freeport Indonesia, Kuala Kencana, Indonesia; 9 Department of Entomology, Faculty of Agriculture, Kasetsart University, Bangkok, Thailand; 10 IES, University of Montpellier, CNRS, Montpellier, France; 11 Cirad, UMR 17, Intertryp, Montpellier, France

**Keywords:** *Anopheles maculatus*, Maculatus Group, Indonesia, Malaria

## Abstract

**Background:**

*Anopheles maculatus* (*s.s.*) is an important vector of malaria in Indonesia. Previously it was considered the only member of the Maculatus Group present in Indonesia. A novel species was recently identified in the Kulon Progo District in Central Java. Until recently, few investigations have been conducted looking at *An. maculatus* genetic diversity in Indonesia, including allopatric island populations.

**Methods:**

Indonesian *An. maculatus* (*s.l.*) samples were collected in several locations in Java, Lesser Sunda Island group, Sumatra and in Kulon Progo (Yogyakarta, central Java) where a novel species has been identified. Samples from a 30-year-old colony of the Kulon Progo population were also included in the analysis. Maximum-likelihood analysis established the phylogenies of the ITS2 (nuclear) and *cox*1 (mitochondrial) markers. Putative times of separation were based on *cox*1 genetic distances.

**Results:**

Two species of the Maculatus Group are present in Indonesia. The novel sibling species is more closely related to *Anopheles dispar* than to *An. maculatus* (*s.s.*). *Anopheles maculatus* (*s.s.*) samples are homogeneous based on the ITS2 sequences. Indonesian samples and *An. dispar* belong to the same *cox*1 maternal lineage and differ from all other known members of the Maculatus Group. Divergence time between the different populations found in Java was estimated using an established *cox*1 mutation rate.

**Conclusions:**

A novel species within the Maculatus Group, most closely related to *An. dispar*, is confirmed present in the Kulon Progo area of Central Java. The divergence of this species from *An. maculatus* (*s.s.*) is explained by the stable refugia in the Kulon Progo area during the quaternary period of intense volcanic activity throughout most of Java. This novel species awaits detailed morphological description before applying a formal species name. For the interim, it is proposed that the Kulon Progo population be designated *An. maculatus* var. *menoreh* to distinguish it from *An. maculatus* (*s.s.*).

**Electronic supplementary material:**

The online version of this article (10.1186/s13071-019-3598-1) contains supplementary material, which is available to authorized users.

## Background

*Anopheles maculatus* (*sensu lato*), in the Neocellia Series [1] of the subgenus *Cellia*, is a widespread species in Asia, ranging from the Indian subcontinent to Southeast Asia and southern China [2–6]. In Indonesia, this species is widely distributed in the western part of the archipelago extending to Weber’s Line, a hypothetical biogeographical separation between Sulawesi and the Maluku Islands chain [7]. *Anopheles maculatus* has been recorded in Sumatra, Java, Kalimantan, Bali, Lesser Sunda Islands including East Timor (Democratic Republic of Timor-Leste), and Sulawesi [8].

Prior to the cytogenetic identification of different chromosomal forms, *An. maculatus* was regarded as a single taxon [9, 10]. Currently, based on phenotypic characteristics, crossmating experiments, cytogenetic and molecular analyses, the Maculatus Group [11] is divided into two subgroups and nine species [3, 10, 12]. The subgroups are differentiated by distinct morphological characters. The Maculatus Subgroup [13] includes *An. maculatus* (*sensu stricto*) Theobald, 1901 and *Anopheles dravidicus* Christophers, 1924, while the Sawadwongporni Subgroup [13] comprises *Anopheles sawadwongporni* Rattanarithikul & Green, 1986 [9], *Anopheles notanandai* Rattanarithikul & Green, 1986 [11] and *Anopheles rampae* Harbach & Somboon, 2011 [14]. The four other species in the group include *Anopheles greeni* Rattanarithikul & Harbach, 1991, *Anopheles dispar* Rattanarithikul & Harbach, 1991, *Anopheles willmori* James, 1903 and *Anopheles pseudowillmori* Theobald, 1910 [12, 15, 16].

The Southeast Asian mainland presents the highest diversity of the Maculatus Group, with seven species present in Thailand [3, 17, 18]. *Anopheles greeni* and *An. dispar* appear restricted (endemic) to the Philippines [15]. Five species are found in China excluding *An. notanandai* and *An. rampae* [3, 19]. In Vietnam, four species [*An. maculatus* (*s.s.*), *An. pseudowillmori*, *An. sawadwongporni* and *An. rampae*] are present [20–22]. Until recently, only *An. maculatus* (*s.l.*) [presumed (*s.s.*)] was reported in Indonesia [8]. A second species has been suspected present in the Kulon Progo District area in Central Java since the late 1990s (MJB, personal communication). This putative, as yet undescribed species was recently reported from material derived from a continuously colonized strain reared [23] over three decades at the Indonesian Ministry of Health Institute for Vector and Reservoir Control Research and Development, a component of the National Institute of Health Research and Development (NIHRD-IVRCRD) and described in this work [24]. *Anopheles maculatus* has long been considered a major malaria vector in West (peninsular) Malaysia [25], and areas of Sumatra and Java, Indonesia [26–30], predominately in rural, forested areas [31]. Numerous instances of natural malaria plasmodia infections in *An. maculatus* have been reported in Indonesia [31]. Infection indices have varied from 2.83% in Kisaran (Sumatra) to 3% in Central Java, 17% in Londut (Sumatra) and 11% in Riau Province (Sumatra) [26, 32]. This species is a major public health concern in the Menoreh Hills region, which includes the Kulon Progo District near the border of Central Java Province and the Special Region of Yogyakarta. It is also reported as a major malaria vector in southern Sumatra (Tenang) [33–36]. Interestingly, although present in Kalimantan, Sulawesi, Bali and the larger islands in the Nusa Tenggara (Lesser Sunda Islands) region, *An. maculatus* has either not been reported as a malaria vector or is an epidemiologically insignificant species in these areas [37].

We analyzed the diversity and phylogeny of *An. maculatus* samples collected in different locations and islands in Indonesia. We also analyzed the relationship of the proposed novel species present in Kulon Progo District and reared at NIHRD-IVRCRD with other members of the Maculatus Group to derive its putative origin.

## Methods

### Mosquito collections and identification

**Fig. 1:**
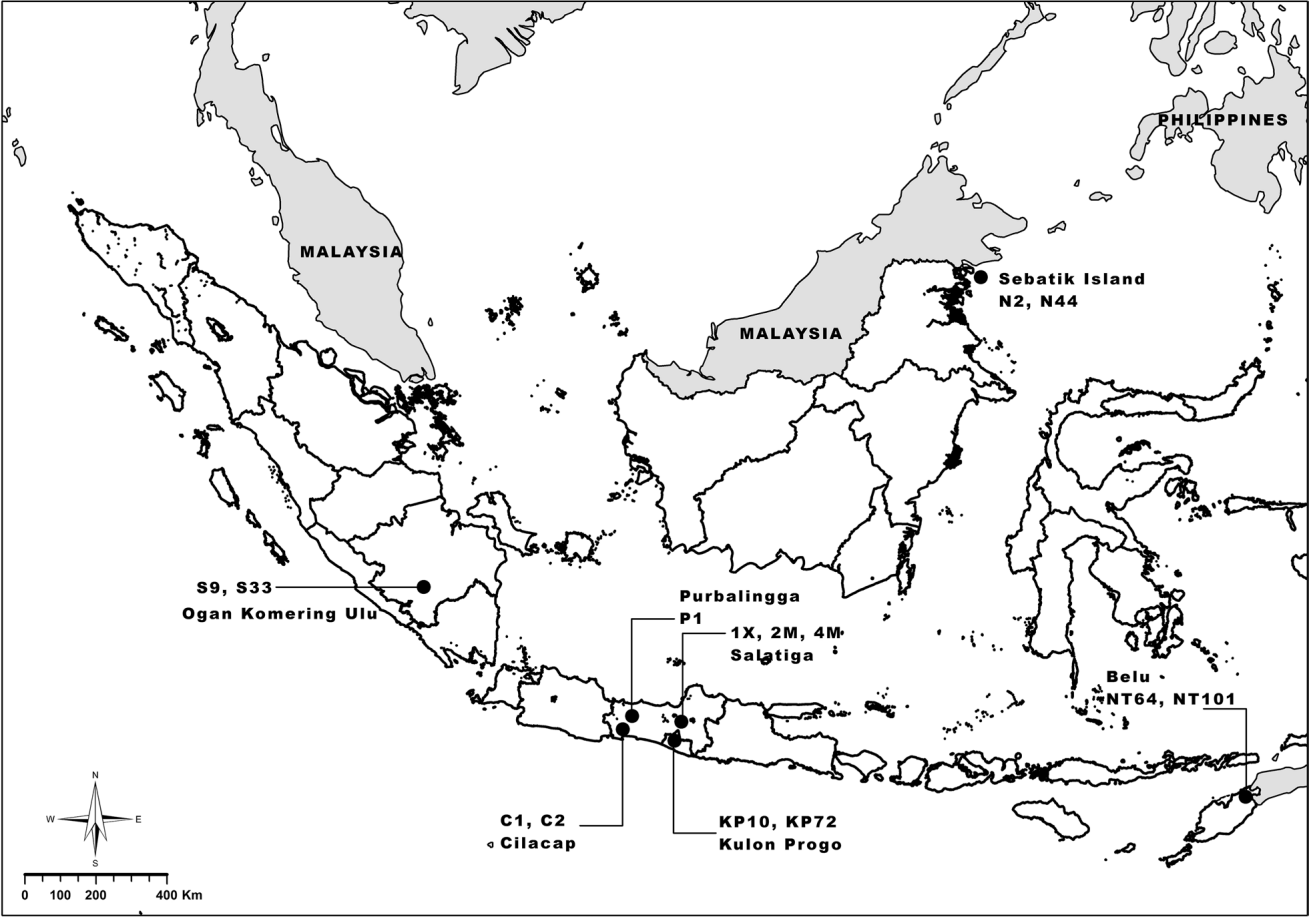
Map of the sampling sites in Indonesia. Each sampling site is indicated as a black spot. The name of sampling sites and samples are indicated. The source of geographical data layers is the Indonesia Geospatial Information Agency who granted the permission and rights to publish this map

Adult mosquitoes were collected from field settings using standard procedures for human-landing and cattle-landing methods [38] in six provinces of Indonesia between 2012 and 2018. Sampling locations included Cilacap, southern Central Java (samples C1 and C2; October 2011), Belu, West Timor, East Nusa Tenggara (samples NT64 and NT 101; November 2011), Ogan Komering Ulu, South Sumatra (samples S9 and S33; October 2011), Sebatik Island, northern Kalimantan (samples N2 and N44; November 2011), Purbalingga, Central Java (sample P1; September 2011), Kulon Progo, Central Java (samples KP10 and KP72; November 2013) and the NIHRD-IVRCRD laboratory, Salatiga (samples 1x, 2M and 4M; October 2018) (Fig. 1; Table 1). *Anopheles maculatus* samples were initially identified using morphological criteria [39]. Mosquitoes were sorted and labeled according to locality and date, and stored in 1.5 ml Eppendorf tubes under dry conditions over silica gel until further analysis [13, 39]. Additionally, a laboratory strain of *An. maculatus* originating from Kulon Progo and under continuous colonization for greater than 30 years at the NIHRD-IVRCRD laboratory in Salatiga, Central Java [23] was compared with more recent field samples from Kulon Progo collected in 2015. To maintain the colony established at IVRCRD Salatiga, wild type material was re-introduced into the laboratory colony in 2003. This re-introduced wild type material was collected in the exact same location as the initial population, i.e. the village of Hargotirto, Kokap subdistrict, Kulon Progo district, Province of Yogyakarta. Representative field-collected specimens are deposited in the Systematics and Reference Laboratory, IVRCRD, Salatiga.

**Table 1:** Sampling localities and specimens of *Anopheles* mosquitoes

Sample code	Location	Ecology	Altitude range (m)	Role as malaria vector	GenBank ID (ITS2)	GenBank ID (*cox*1)
P1	Purbalingga, Java	Wet rice field, plantation	250–329	Yes	MK656100	MK683475
C1	Cilacap, Java	Secondary forest, plantation, wet rice field	300–348	No	MK656095	MK683467
C2	Cilacap, Java	Secondary forest, plantation, wet rice field	300–348	No	MK656096	MK683468
KP10	Kulon Progo Java	Secondary forest, wet rice field	300–1000	Yes	MK659792	MK683471
KP72	Kulon Progo Java	Secondary forest, wet rice field	300–1000	Yes	MK659780	MK683472
1x	Insectary laboratory IVRCRD Salatiga (origin Kulon Progo)	Laboratory conditions	700	Yes	MK659773	MK683464
2M	Insectary laboratory IVRCRD Salatiga (origin Kulon Progo)	Laboratory conditions	700	Yes	MK675654	MK683465
4M	Insectary laboratory IVRCRD Salatiga (origin Kulon Progo)	Laboratory conditions	700	Yes	MK675653	MK683466
NT64	Belu, East Nusa Tenggara	Secondary forest, wet rice field	150–215	Yes	MK659796	MK683473
NT101	Belu, East Nusa Tenggara	Secondary forest, wet rice field	150–215	Yes	MK659794	MK683474
S9	Ogan Komering Ulu Sumatra	Coffee and rubber plantations	800–892	Yes	MK659795	MK683476
S33	Ogan Komering Ulu Sumatra	Coffee and rubber plantations	800–892	Yes	MK659793	MK683477
N2	Sebatik Island Kalimantan	Coconut, palm oil, coffee and cacao plantations	150–218	Yes	MK659798	MK683469
N44	Sebatik Island Kalimantan	Coconut, palm oil, coffee and cacao plantations	150–218	Yes	MK659797	MK683470

### DNA extraction, amplification and sequencing

DNA was extracted from the legs of each mosquito using a DNeasy® Blood & Tissue Kit (Qiagen, Hilden, Germany) with modification based on the manufacturer’s protocol. The amplification of ITS2 was performed with primers ITS2a (5′-TGT GAA CTG CAG GAC ACA T-3′) and ITS2b (5′-TAT GCT TAA ATT CAG GGG GT-3′) [39]. *cox*1 was amplified using the primers CI-N-2087 (5′-AAT TTC GGT CAG TTA ATA ATA TAG-3′) and TY-J-1460 (5′-TAC AAT TTA TCG CCT AAA CTT CAG CC-3′). PCR reactions were carried out using GoTaq® Green Master Mix (Promega, Madison, WI, USA). PCR thermocycling conditions for ITS2 were as follows: 94 °C for 10 min; followed by 40 cycles of denaturation at 94 °C for 1 min, annealing at 56 °C for 45 s and elongation at 72 °C for 1 min; followed by a final extention step at 72 °C for 10 min. For amplification of the *cox*1 gene, the following conditions were used: initial denaturation at 94 °C for 1 min followed by five cycles of 94 °C for 30 s, 45 °C for 40 s and 72 °C for 1 min; this was then followed by 35 cycles of 94 °C for 30 s, 55 °C for 40 s and 72 °C for 1 min, and by a final extention step at 72 °C for 10 min [40]. The amplified PCR products were separated by 1.5% agarose gel electrophoresis and vizualized by SYBR® safe DNA gel stain (Invitrogen, Carlsbad, CA, USA). A 100-bp DNA ladder was used for calculating the size of the PCR products. Amplification products were purified using Applied Biosystems ExoSAP-IT™ (Thermo Fisher Scientific, Vilnius, Lithuania). Cycle sequencing was performed using the primers listed above and an Applied Biosystems BigDye™ Terminator v.3.1 Cycle Sequencing Kit (Life Technologies Cooperation, Austin, TX, USA). To remove unicorporated BigDye® terminators and salts, cycle sequencing products were purified using a BigDye® Xterminator Purification Kit (Life technologies, Bedford, MA, USA). Sequence data were obtained using a DNA sequencer (Applied Biosystems® 3500 Genetic Analyzer) and analyzed using the Sequencing Analysis 6 program (Applied Biosystems).

### Sequence analysis

Sequences were edited using Sequencing Analysis v.5.2 (Applied Biosystems). Sequences were aligned with MUSCLE using SeaView v.4.7 [41] and Mega X [42]. Phylogenetic trees were constructed with the maximum likelihood (ML) method and the Kimura-2 (K80) evolutionary model in Mega X. To assess the ML tree reliability, bootstraps were tested with 1000 replicates. To estimate the evolutionary divergence between sequences, genetic distances were analyzed by pairwise distance (p-distance) methods [43] in Mega X. Divergence time was calculated based on previously reported estimates giving 1 million years (Myr) for 2.3% difference [44, 45]. Sequences are deposited in GenBank under the following accession numbers: ITS2: N2 (MK659798), N44 (MK659797), S9 (MK659795), S33 (MK659793), NT64 (MK659796), NT101 (MK659794), KP10 (MK659792), KP72 (MK659780), 1x (MK659773), 2M (MK675654), 4M (MK675653), P1 (MK656100), C1 (MK656095) and C2 (MK656096); *cox*1: N2 (MK683469), N44 (MK683470), S9 (MK683476), S33 (MK683477), NT64 (MK683473), NT101 (MK683474), KP10 (MK683471), KP72 (MK683472), 1x (MK683464), 2M (MK683465), 4M (MK683466), P1 (MK683475), C1 (MK683467) and C2 (MK683468).

## Results

### ITS2 diversity and phylogeny of *Anopheles maculatus*

**Fig. 2:**
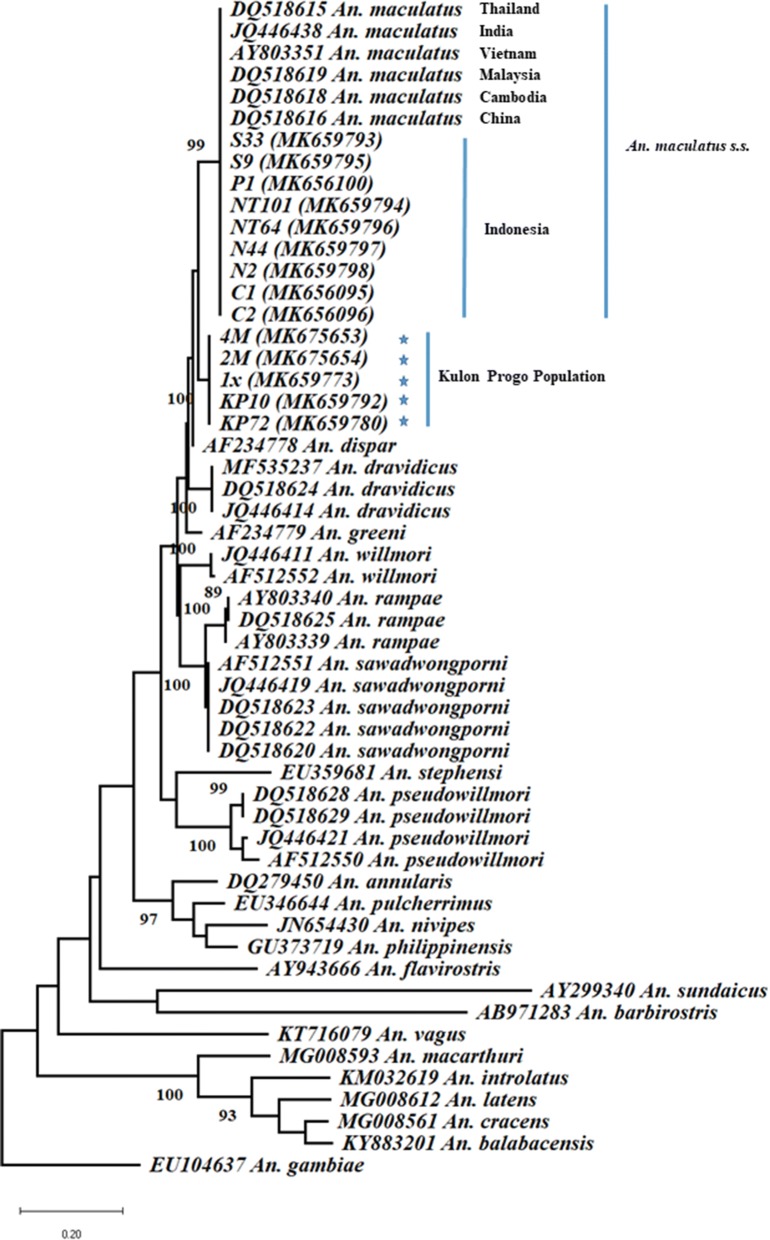
Phylogenetic analysis of the ITS2 sequences. Kulon Progo samples are identified with blue stars. The phylogenetic tree was constructed using the maximum likelihood (ML) method and the Kimura-2 (K80) evolutionary model in Mega X. To assess the ML tree reliability, bootstraps were tested at 1000 replicates

The comparative analysis of the ITS2 sequences of all *An. maculatus* samples and of available reference sequences from other members of the Maculatus Group and select other *Anopheles* species present in Indonesia indicates that two populations of *An. maculatus* are present in Indonesia. Samples of *An. maculatus* coming from Purbalingga (P1), Cilacap (C1, C2), Belu (NT64, NT101), Sebatik Island (N2, N44) and Ogan Komering Ulu (S9, S33) displayed 100% genetic similarity and were also 100% identical to *An. maculatus* sequences from the mainland Asian continent (Fig. 2, Additional file 1: Table S1). The GenBank *An. maculatus* sequences used as reference corresponded to mosquitoes isolated in India (JQ446438), Thailand (DQ518615), Vietnam (AY803351), Malaysia (DQ518619), Cambodia (DQ518618) and China (DQ518616). For the nuclear ribosomal ITS2 sequence, the similarity between all *An. maculatus* reference sequences and sequences from samples P1, C1, C2, NT64, NT101, N2, N44, S9 and S33 indicated a high conservation and genetic homogeneity regardless of distribution and geographical distance (Fig. 2). There was also no difference between samples from the continental Asian land mass and island groups. Conversely, the samples KP10, KP72, 1x, 2M and 4M isolated from Kulon Progo did not cluster with the continental *An. maculatus* sequences producing a separate, genetically distinct and homogeneous group more closely related to *An. dispar*. The sequences 1x, 2M and 4M, which correspond to a laboratory strain of *An. maculatus* collected decades ago in Kulon Progo, were identical to those samples collected for this work, i.e. KP10 and KP72, indicating strong genetic stability after years of continuous colonization.

This phylogenetic analysis indicates that although separated into two different genetic aggregates (groups), collectively the Indonesian samples belong to the Maculatus Group. With respect to genetic distances, the samples displayed two ranges of distances depending on the group considered (Additional file 1: Table S1). The Indonesian *An. maculatus* group, i.e. samples P1, C1, C2, NT64, NT101, S9, S33, N2 and N44, showed no genetic distance with the continental *An. maculatus* reference sequences. The ITS2 sequence appears entirely conserved. The genetic distance within the Maculatus Group ranged between 2.7–20.8%, while the genetic distance of the *An. maculatus* sequences from other group members ranged between 5–16.5% (Additional file 1: Table S1). The Kulon Progo samples (KP10, KP72, 1x, 2M and 4M) displayed no (0%) internal group distance and a 5.5–5.8% distance with the other *An. maculatus* sequences. Comparison with other members, the Kulon Progo sequences displayed distances of 3%, 6.5%, 7.7–8, 8%, 10.4–10.9%, 15.5–17% and 10.9% with *An. dispar*, *An. greeni*, *An. dravidicus*, *An. sawadwongporni*, *An. willmori*, *An. pseudowillmori* and *An. rampae*, respectively (Additional file 1: Table S1). The alignment of the Kulon Progo ITS2 sequences with *An. maculatus* (*s.s.*) from Indonesia, *An. maculatus* (*s.s.*) from mainland Asia, and *An. dispar* is provided in Additional file 2: Figure S1.

### *cox*1 diversity and phylogeny of *Anopheles maculatus*

**Fig. 3:**
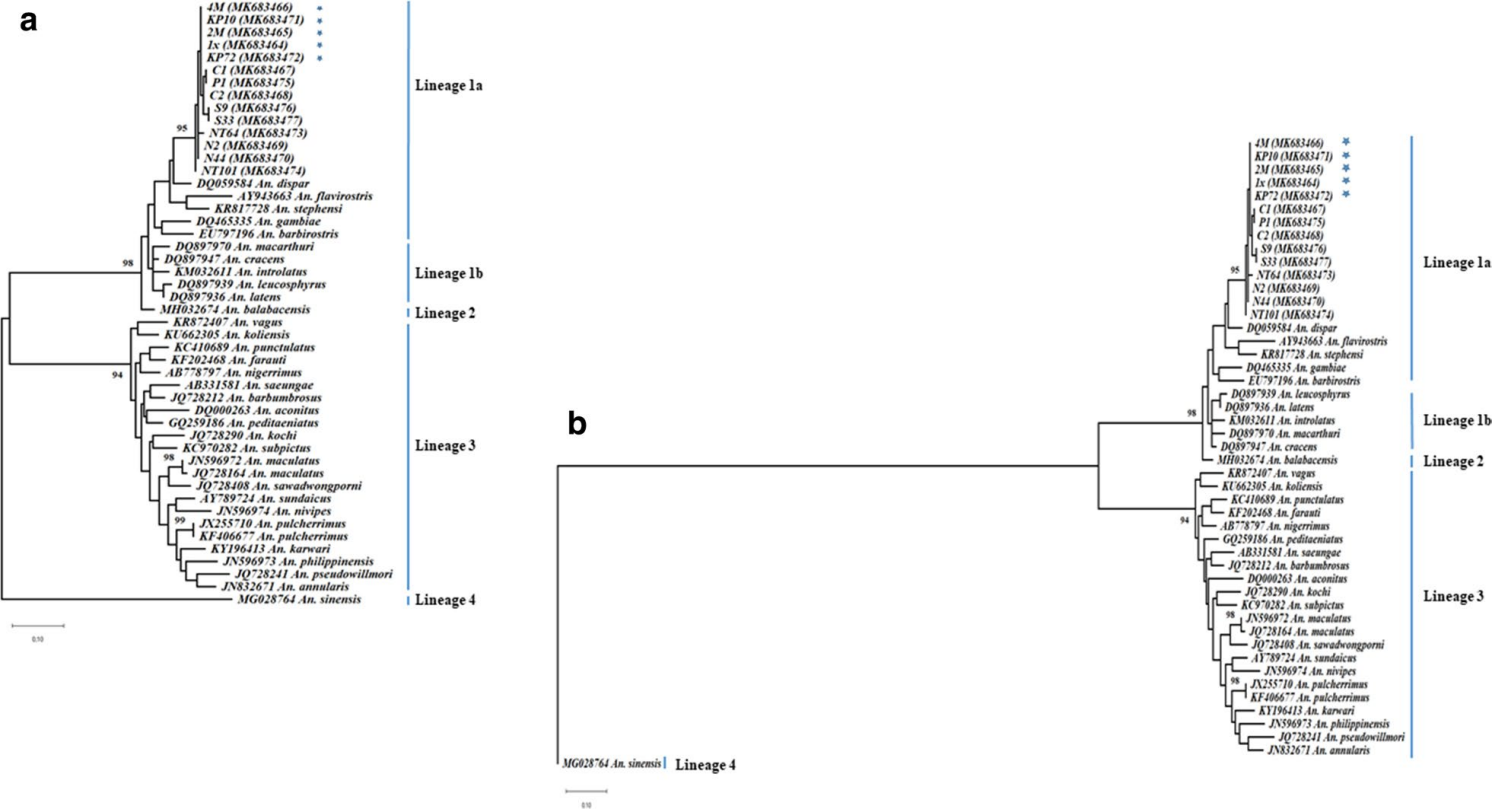
Phylogenetic analysis of the *cox*1 sequences. **a** Midpoint rooted tree. **b** Tree rooted using *Anopheles sinensis* as outgroup. Kulon Progo samples are identified with stars. Phylogenetic trees were constructed with the maximum likelihood (ML) method and the Kimura-2 (K80) evolutionary model in Mega X. To assess the ML tree reliability, bootstraps were tested with 1000 replicates

The comparative analysis of the mitochondrial *cox*1 sequences, indicative of the maternal lineage, showed that all the samples and reference sequences belonged to four genetically distinct and separated lineages, Lineage 1 being separated into two sublineages (Fig. 3a). Lineage 4 comprised only *Anopheles sinensis* (subgenus *Anopheles*, Hyrcanus Group) and was therefore used as outgroup for the rooted tree (Fig. 3b). Lineage 1a comprised *Anopheles stephensi*, *Anopheles flavirostris*, *An. dispar* (a Maculatus Group member), all the Indonesian samples including the Kulon Progo population, *An. gambiae* and *Anopheles barbirostris* (Clade I); while Lineage 1b included three out four species of the Leucosphyrus Complex, along with *Anopheles macarthuri*, a member of the Riparis Subgroup, and *Anopheles cracens*, a member of the Dirus Complex, all five belonging to the Leucosphyrus Group [14]. Lineage 2 comprised only *An. balabacensis*, the fourth species of the Leucosphyrus Complex. Lineage 3 comprised all of the *An. maculatus* reference samples and members of the Maculatus Group available in GenBank, excluding *An. dispar* and the Indonesian *An. maculatus* samples. Lineage 3 and Lineage 4 branched directly on the root; whereas, Lineage 1a and Lineage 1b were further separated by a bootstrap of 99. The Indonesian *An. maculatus* sequences within Lineage 1a grouping displayed some internal genetic variability. All Kulon Progo samples examined were identical, while genetic distances of up to 2.6% were observed with *An. maculatus* (*s.s.*) from Indonesian (Additional file 3: Table S2). With respect to the other members of Lineage 1a, the closest species was *An. dispar* with a percentage of divergence ranging between 7.8–8.4% depending on the sample. The divergence of the Indonesian samples with *An. flavirostris* (Minimus Subgroup) ranged between 12.2–13.5%, while *An. stephensi* (Neocellia Series) ranged between 10.4–12.2% (Additional file 3: Table S2). The alignment of the *cox*1 sequences of the Kulon Progo samples, *An. maculatus* (*s.s.*) from Indonesia, *An. maculatus* (*s.s.*) from mainland Asia, and *An. dispar* is provided in Additional file 4: Figure S2.

### Time of divergence

The time of divergence was calculated based on previously reported estimates of the variation of the *cox*1 gene in the genus *Anopheles* where 2.3% of divergence is estimated to correspond to 1 million years (Myrs) [42, 43]. The estimated time of divergence of *An. maculatus* (*s.s.*) from the Kulon Progo population and from *An. dispar* was estimated at between 26–26.2 Myrs, and between 30.2–30.9 Myrs, respectively, which corresponds to the Oligocene Epoch. The divergence of the Kulon Progo population from the other members of the Kulon Progo maternal lineage, i.e. *An. dispar*, *An. flavirostris* and *An. stephensi*, is dated 3.4 (Pliocene Epoch), 5.3 (Miocene Epoch) and 5.04 Myrs (Pliocene), respectively. The other Indonesian *An. maculatus* sequences displayed the same separation time with *An. dispar* as the Kulon Progo population with the exception of NT101 (Belu, East Nusa Tenggara), C1 (Cilacap, Central Java) and P1 (Purbalingga, Central Java), indicating separation around 3.13 and 3.65 Myrs ago (Pliocene), respectively. The separation of the Kulon Progo population from the other Indonesian *An. maculatus* samples was dated 0.65 (NT64, S9, S33), 0.43 (NT101, C1, P1) and 0.22 Myrs (C2, N2, N44), all corresponding to the latter part of the Pleistocene Epoch.

## Discussion

*Anopheles maculatus* (*s.s.*) was previously believed to be the only member of the Maculatus Group present in Indonesia, a species regarded as an important vector of malaria in certain localities [6]. This Asian group is a diverse assemblage with at least nine described species, five of which fall into two subgroups [3, 10, 12]. Investigating the diversity of *An. maculatus* in Indonesia was therefore a prerequisite for a better understanding of the distribution, bionomics and variations in vector capacity over its wide geographical range. The investigation reported herein provides several conclusions. First, there is definitive evidence of at least two species within the Maculatus Group in Indonesia, confirming a recent report by Ali et al. [24], which raises the number of species to ten (none of which are nominal taxon) within the Maculatus Group. Secondly, *An. maculatus* (*s.s.*) appears genetically homogeneous throughout its geographical range in Asia. Thirdly, members of the group in Indonesia differ by maternal origin from all other members, including *An. maculatus* (*s.s.*) from mainland Asia, with the lone exception of *An. dispar.*

The Kulon Progo population has been suspected as a distinct species within the Maculatus Group based on unpublished work spanning several decades (MJB, personal comm). This population was recently proposed as a different species based on selected morphological characters and genetic (ITS2 and *cox*2) sequences [24]. The ITS2 phylogenetic analysis in the present study confirmed that the Kulon Progo population and all other known *An. maculatus* sequences analyzed in Indonesia are members of the Maculatus Group, yet they also comprise genetically distinct groups. ITS2 is not considered a good intraspecific marker due to its low evolution rate and high conservation; however, it is a good marker at the species level showing clear discrimination indicative of species separation [46–50]. The phylogenetic distance between the ITS2 sequences of the Kulon Progo population and other *An. maculatus* sample sequences both Indonesia and mainland Asia included in the analysis ranged between 5.5–5.8%. This is greater than the ITS2 distances displayed by other groups of closely related *Anopheles* species. For example, two sibling species in the *Anopheles farauti* complex (an assemblage of 8 species) differ by only 4.0% [51], while *An. greeni* and *An. dispar* (Maculatus Group) also differ by 4.0% [52]. In Africa, five species within the *An. gambiae* complex show intraspecific differences ranging between 0.4–1.6% [53], while two members of the *An. dirus* complex, *An. dirus* (*s.s.*) (formerly species A) and *An. baimaii* (species D), display 5.4% genetic distance [54]. The Kulon Progo population was shown to be morphologically distinct from *An. maculatus* (*s.s.*) while cross-mating experiments generated partially sterile hybrids [24]. The combined evidence confirms that the Kulon Progo population is a distinct species and one that likely extends throughout the greater Menoreh Hill region in central Java. Until a formal morphological description can be made, it is hereby proposed that the Kulon Progo population be designated an infrasubspecific entity, *An. maculatus* var. *menoreh*, in reference to its region of origin and to distinguish it from *An. maculatus* (*s.s.*).

The two Indonesian members of the Maculatus Group and *An. dispar* belong to the same mitochondrial lineage and differ from that of all other known members of the group outside Indonesia. Collectively, these data demonstrate the occurrence in Indonesia of an introgression of the *An. maculatus* (*s.s.*) chromosomal genome from continental to insular populations. A similar phenomenon of introgression has been demonstrated for *Anopheles sundaicus* complex in Southeast Asia [44]. Introgression is a key adaptive mechanism of *Anopheles* mosquitoes to exist in various environments [44, 55], and well described in the *Anopheles gambiae* complex [56, 57].

The Pleistocene Epoch (2.58 Myrs to 11,700 years ago) is believed to have played a key role in the distribution of *Anopheles* mosquitoes in Southeast Asia [46, 58–60]. The period was characterized by a series of glaciation and inter-glaciation periods, which generated dramatic climatic changes and large variations in sea level [61, 62]. During glaciation periods, islands west of the Wallace’s Line were interconnected on the same land mass known as the Sunda Shelf [63–65]; whereas, during inter-glaciation events the rainforest environments expanded, thus providing more favorable habitats for *Anopheles* mosquitoes while island landmasses remained isolated. Sulawesi, the eastern Lesser Sunda and Maluku island chains, and western New Guinea Island were isolated and separated by sea from the western half of the Indonesian archipelago, while the Philippines followed a different biogeographical evolution. Palawan Island (western Philippines) was then connected to the Sunda Shelf but later separated and collided with the mobile belt of the Philippine archipelago. This geological history induced by shifts in climate is considered to have greatly influenced the current structural diversity of *Anopheles* populations in Southeast Asia and the evolution of present-day species complexes through successive genetic expansions and bottlenecks [66].

Based on the molecular evidence, the Kulon Progo population appears more closely related to *An. dispar*, a species that appears confined to the northern Philippines. The most parsimonious way to explain this geographical discrepancy is that their common ancestors gradually moved from continental Asia to the current island territories during the Oligocene, which corresponds to the calculated separation of the Kulon Progo lineage from the continental *An. maculatus* (*s.s.*) lineage (between 23 and 26.4 Myrs). A movement of *An. maculatus* from the continent appears to have occurred before 3.4 Myrs ago (between the late Oligocene and early Pliocene epochs), the calculated time of separation between *An. dispar* and the Kulon Progo population. This event led to introgression of the *An. maculatus* chromosomal genome into at least a portion of the maternal lineage identified as Lineage 1. During the Pliocene (3.4 Myrs ago), the ancestor of *An. dispar* was separated from the main introgressed population, likely the result of the tectonic shift of Palawan Island towards the current Philippine archipelago. During the Pleistocene, increased volcanism occurred in central and eastern Java but the Kulon Progo area was naturally spared from the surrounding destruction and served as a relic forest refuge [67]. This isolation event occurred between 0.22 and 0.65 Myrs, which corresponds to the calculated separation time between the Kulon Progo population and the other Indonesian archipelagic *An. maculatus* populations. During the late Pleistocene period (200,000 to 11,700 years ago), at least one other species invasion and introgression by continental *An. maculatus* appears to have occurred which generated the current Indonesian populations of *An. maculatus* (*s.s.*). This timescale is in agreement with that calculated for the movements of populations and introgression detected in *An. sundaicus* in Southeast Asia [44, 45].

## Conclusions

*Anopheles maculatus*, along with *Anopheles balabacensis*, is the main malaria vector species occuring in the Kulon Progo area and the greater Menoreh region [33–37, 68–71]. The evidence presented here confirms that the Kulon Progo population is a distinct species and one that likely extends throughout the greater Menoreh Hill region in central Java. There are now two recognized members of the Maculatus Group present in Indonesia. However, a detailed morphological description of this novel species is required to establish a new nominal taxon. To distinguish it from *An. maculatus* (s.s.), in the interim it is hereby proposed an infrasubspecific entity (‘variety’), *An. maculatus* var. *menoreh.*

## Additional files

**Additional file 1: Table S1.** Pairwise genetic distance of ITS2 sequences. Genetic distances were calculated with the Kimura 2 parameters using Mega X.

**Additional file 2: Figure S1.** Alignment of ITS2 sequences. Alignment performed using Seaview v.4.7 with MUSCLE program for multialignment.

**Additional file 3: Table S2.** Pairwise genetic distance of *cox*1 sequences. Genetic distances were calculated with the Kimura 2 parameters using Mega X.

**Additional file 4: Figure S2.** Alignment of *cox*1 gene sequences. Alignment performed using Seaview v.4.7 with MUSCLE program for multialignment.

## Data Availability

Data supporting the conclusions of this article are included within the article and its additional files. Raw data are available from the corresponding author upon reasonable request. ITS2 sequences are deposited under accession numbers: N2 (MK659798), N44 (MK659797), S9 (MK659795), S33 (MK659793), NT64 (MK659796), NT101 (MK659794), KP10 (MK659792), KP72 (MK659780), 1x (MK659773), 2M (MK675654), 4M (MK675653), P1 (MK656100), C1 (MK656095) and C2 (MK656096). *cox*1 sequences are deposited under accession numbers: N2 (MK683469), N44 (MK683470), S9 (MK683476), S33 (MK683477), NT64 (MK683473), NT101 (MK683474), KP10 (MK683471), KP72 (MK683472), 1x (MK683464), 2M (MK683465), 4M (MK683466), P1 (MK683475), C1 (MK683467) and C2 (MK683468).

## Abbreviations

ITS2: internal transcribed spacer 2
*cox*1: cytochrome oxidase subunit I
ML: maximum likelihood
