# Supplementary material for: Genetic homogeneity of Anopheles maculatus in Indonesia and origin of a novel species present in Central Java
Source: Parasit Vectors. 2019 Jul 15;12:351. doi: 10.1186/s13071-019-3598-1 (PMC6631912; doi:10.1186/s13071-019-3598-1)
Supplement: Supplementary file 2 — Additional file 2: Figure S1. Alignment of ITS2 sequences. Alignment performed using Seaview v.4.7 with MUSCLE program for multialignment. [file 13071_2019_3598_MOESM2_ESM.pdf]

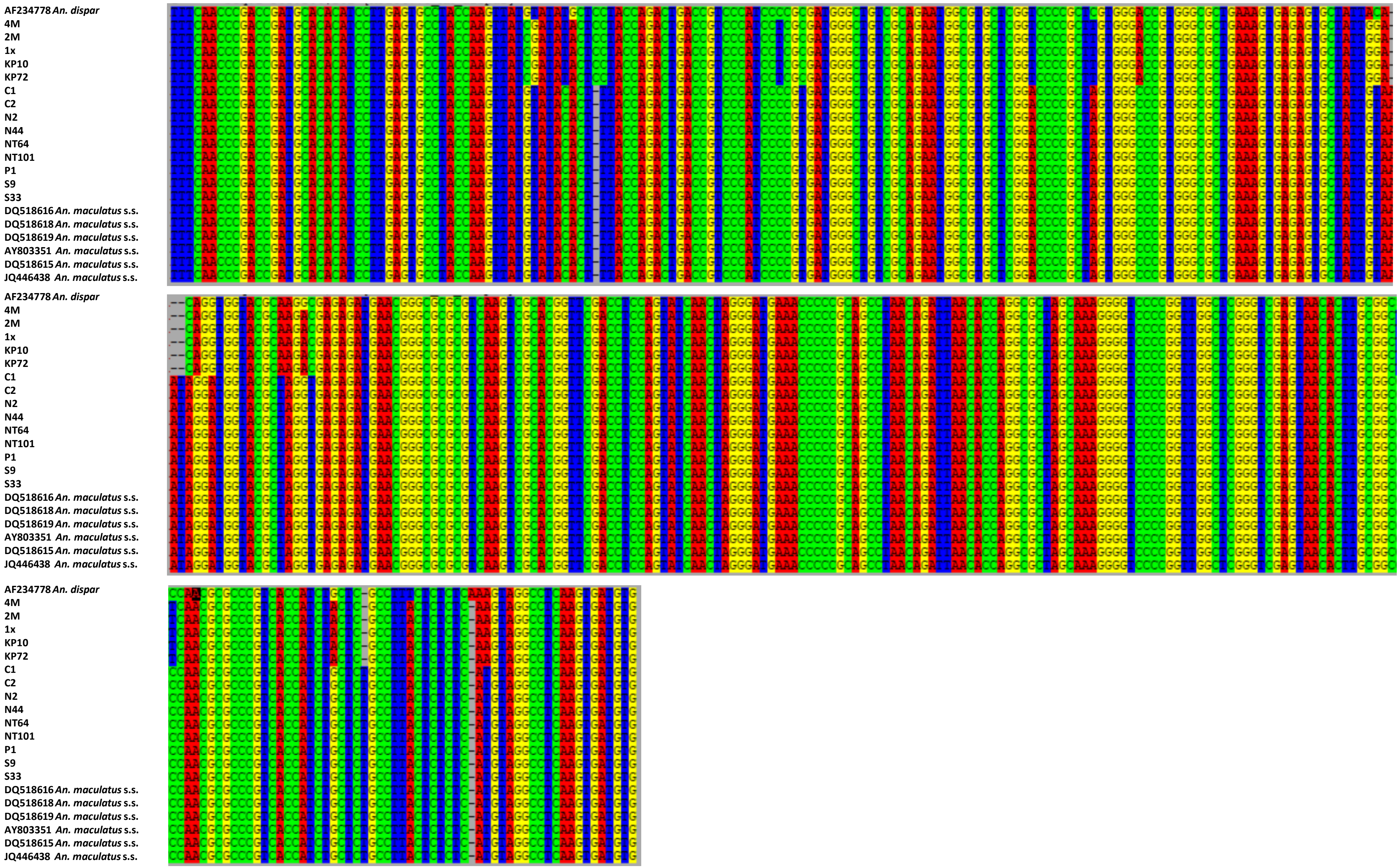
AF234778 An. dispar
4M
2M
1x
KP10
KP72
C1
C2
N2
N44
NT64
NT101
P1
S9
S33
DQ518616 An. maculatus s.s.
DQ518618 An. maculatus s.s.
DQ518619 An. maculatus s.s.
AY803351 An. maculatus s.s.
DQ518615 An. maculatus s.s.
JQ446438 An. maculatus s.s.
AF234778 An. dispar
4M
2M
1x
KP10
KP72
C1
C2
N2
N44
NT64
NT101
P1
S9
S33
DQ518616 An. maculatus s.s.
DQ518618 An. maculatus s.s.
DQ518619 An. maculatus s.s.
AY803351 An. maculatus s.s.
DQ518615 An. maculatus s.s.
JQ446438 An. maculatus s.s.
AF234778 An. dispar
4M
2M
1x
KP10
KP72
C1
C2
N2
N44
NT64
NT101
P1
S9
S33
DQ518616 An. maculatus s.s.
DQ518618 An. maculatus s.s.
DQ518619 An. maculatus s.s.
AY803351 An. maculatus s.s.
DQ518615 An. maculatus s.s.
JQ446438 An. maculatus s.s.
