# Supplementary material for: Genetic homogeneity of Anopheles maculatus in Indonesia and origin of a novel species present in Central Java
Source: Parasit Vectors. 2019 Jul 15;12:351. doi: 10.1186/s13071-019-3598-1 (PMC6631912; doi:10.1186/s13071-019-3598-1)
Supplement: Supplementary file 4 — Additional file 4: Figure S2. Alignment of cox1 gene sequences. Alignment performed using Seaview v.4.7 with MUSCLE program for multialignment. [file 13071_2019_3598_MOESM4_ESM.pdf]

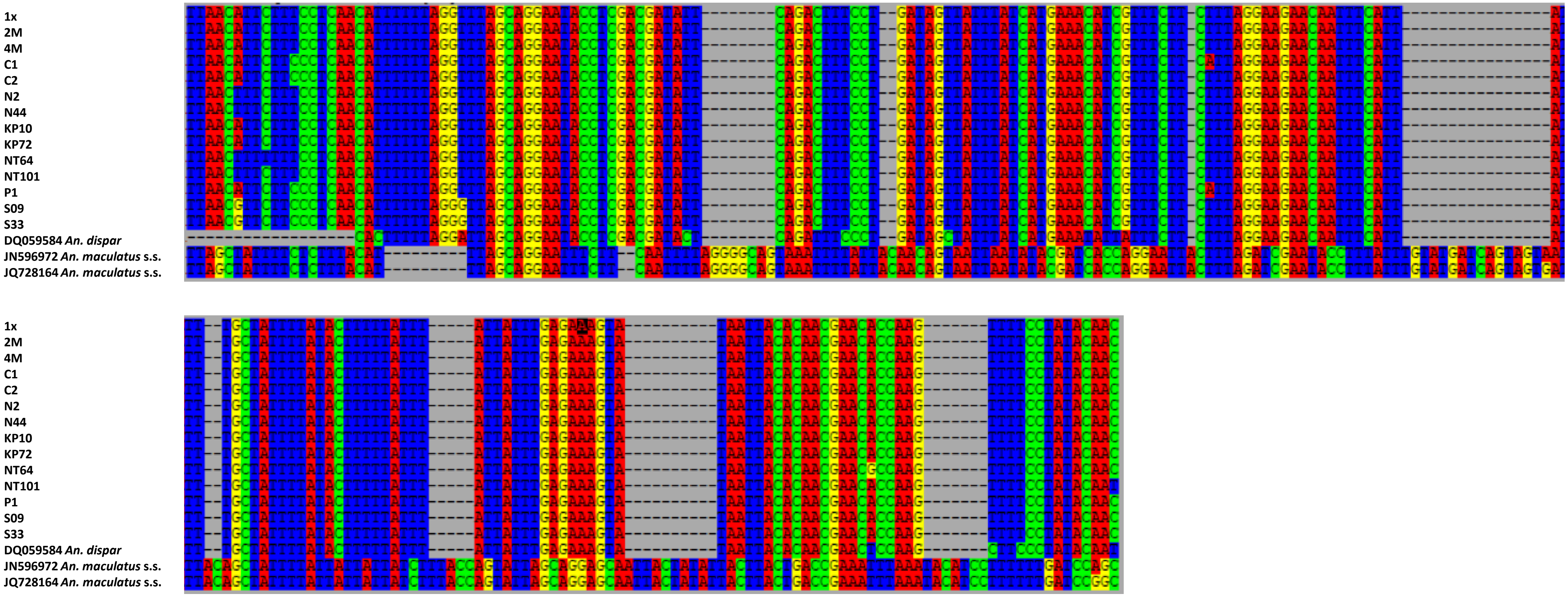
1x
2M
4M
C1
C2
N2
N44
KP10
KP72
NT64
NT101
P1
S09
S33
DQ059584 An. dispar
JN596972 An. maculatus s.s.
JQ728164 An. maculatus s.s.
1x
2M
4M
C1
C2
N2
N44
KP10
KP72
NT64
NT101
P1
S09
S33
DQ059584 An. dispar
JN596972 An. maculatus s.s.
JQ728164 An. maculatus s.s.
